# Epilepsy Seizures Prediction Based on Nonlinear Features of EEG Signal and Gradient Boosting Decision Tree

**DOI:** 10.3390/ijerph191811326

**Published:** 2022-09-09

**Authors:** Xin Xu, Maokun Lin, Tingting Xu

**Affiliations:** School of Communications and Information Engineering, Nanjing University of Posts and Telecommunications, Nanjing 210003, China

**Keywords:** EEG, epilepsy, seizure prediction, nonlinear features, GBDT, ensemble learning

## Abstract

Epilepsy is a common neurological disorder with sudden and recurrent seizures. Early prediction of seizures and effective intervention can significantly reduce the harm suffered by patients. In this paper, a method based on nonlinear features of EEG signal and gradient boosting decision tree (GBDT) is proposed for early prediction of epilepsy seizures. First, the EEG signals were divided into two categories: those that had seizures onset over a period of time (represented by InT) and those that did not. Second, the noise in the EEG was removed using complementary ensemble empirical mode decomposition (CEEMD) and wavelet threshold denoising. Third, the nonlinear features of the two categories of EEG were extracted, including approximate entropy, sample entropy, permutation entropy, spectral entropy and wavelet entropy. Fourth, a GBDT classifier with random forest as the initial result was designed to distinguish the two categories of EEG. Fifth, a two-step “k of n” method was used to reduce the number of false alarms. The proposed method was evaluated on 13 patients’ EEG data from the CHB-MIT Scalp EEG Database. Based on ten-fold cross validation, the average accuracy was 91.76% when the InT was taken at 30 min, and 38 out of 39 seizures were successfully predicted. When the InT was taken for 40 min, the average accuracy was 92.50% and all 42 seizures selected were successfully predicted. The results indicate the effectiveness of the proposed method for predicting epilepsy seizures.

## 1. Introduction

Epilepsy is one of the most common neurological disorders in humans, affecting approximately 60 million people worldwide who are unable to live normally [1]. Approximately 70–80% of epilepsy patients are well controlled with medication or surgery, but about 20–30% of patients are still not effectively treated [2]. During seizures, patients experience loss of consciousness, involuntary convulsions and mental abnormalities. If a patient has a seizure while exercising or driving, it is likely to cause further harm and place a heavy burden on the patient and their family [3]. Therefore, if seizures can be predicted and interfered with in a timely manner, it can reduce the harm caused by seizures and improve the quality of life for patients.

Electroencephalographic (EEG) signals directly record the electrical activity in the cerebral cortex and contain a wealth of information about brain activity, which are widely used in the fields of brain disease diagnosis and medical rehabilitation. Depending on the method of acquisition, there are two types of EEG: intracranial EEG (iEEG) and scalp EEG (sEEG). Although sEEG is susceptible to interference from external factors, its non-invasive and convenient acquisition properties make it one of the most important tools in epilepsy diagnosis and treatment. With the continuous development of signal processing and artificial intelligence (AI), an increasing number of researchers have been working on the early prediction of epilepsy seizures using EEG, with some success.

At present, methods for epilepsy seizures prediction using EEG can be divided into two categories: threshold-based prediction methods and classification-based methods [3]. The former involves identifying patterns of changes in certain features of the EEG prior to seizures and using this pattern to determine whether a seizure is likely to occur. Salvatierra et al. [4] calculated the spectral power ratio (SPR) of the EEG in the hour before a seizure and plotted the SPR curve to predict seizures using the pattern of change in SPR. The method was evaluated on the CHB-MIT Scalp EEG Database with an optimal prediction time of 60 min and a prediction success rate of 100%. The threshold-based prediction method is intuitive and easy to understand, but the predictive effect of the same threshold varies greatly from patient to patient, making it difficult to establish stable models. In contrast, classification-based methods are more stable and effective, and are the main methods used for seizures prediction. This type of method extracts several features from the interictal and pre-ictal EEG signals, and uses machine learning or deep learning to identify the pre-ictal EEG to achieve seizures prediction. Zhang et al. [5] decomposed the patient’s EEG into eight frequency bands, extracted features for each band using a common spatial pattern (CSP), and then used a convolutional neural network (CNN) to classify the pre-ictal and interictal EEGs. This method obtained an average sensitivity of 0.92, an average accuracy of 0.90, and an average false alarm rate of 0.12/h. Usman et al. [6] combined handcrafted EEG features (mean, kurtosis, approximate entropy, etc.) with features automatically extracted using a three-layer CNN, and designed an ensemble classifier with SVM, CNN, and LSTM as base classifiers for EEG classification, which achieved a sensitivity of 96.28% and an average prediction time of 33 min. As patients with epilepsy spend most of their time in an interictal state, the amount of interictal data is much larger than the amount of pre-ictal data. Therefore, classification-based methods often suffer from class imbalance problems, which affect the performance of the model. Slicing and re-splicing EEG data or using generative adversarial networks (GAN) can increase the amount of pre-ictal data and, thus, mitigate the impact of the imbalanced class problem [6,7].

In this work, the epileptic EEG signal was divided into two categories; category A has seizures after a time interval, while category B has no seizures after a time interval. This interval is referred to as the interval time (InT) and can be either 30 or 40 min. The data in category B were taken from an interval of 4 consecutive hours without a seizure and had the same amount of data as the data in category A. This approach addressed the class imbalance and made full use of the patient’s EEG data. The two categories of EEG signals were first pre-processed by complementary ensemble empirical mode decomposition (CEEMD) and wavelet threshold denoising. Then, the approximate entropy, sample entropy, permutation entropy, spectral entropy and wavelet entropy were extracted as features and classified using a gradient boosting decision tree (GBDT) with random forest as the initial result.

The rest of this paper is organized as follows: Section 2 describes the dataset, pre-processing, feature extraction, classification and post-processing methods, Section 3 presents the results, and the discussion and conclusions are in Section 4 and Section 5 respectively.

## 2. Materials and Methods

### 2.1. Datasets

The EEG data used in this paper were obtained from the CHB-MIT Scalp EEG Database [7], which contains sEEG data from 22 epilepsy patients aged 3–22. Each patient containsbetween 9 and 42 EEG data files, recorded at a sampling rate of 256 Hz and 16 bit resolution, with electrodes named and placed according to the international 10–20 system. An annotation file in the dataset described the start and end time of each seizure. Hardware limitations resulted in gaps between consecutively-numbered EEG files, during which the signals were not recorded; in most cases, the gaps were 10 s or less.

### 2.2. Data Segmentation

For each patient, EEG data were divided into two categories, A and B. For a seizure, the period before the onset of the seizure was defined as the interval time (InT), and the EEG data from 10 min before the InT was included as part of the category A data. It should be noted that if the time interval between two seizures was short, then the category A data for the second seizure might contain the ictal or pre-ictal EEG of the first seizure, which would make the category A data ‘impure’. Therefore, seizures that were the subjects of this study were not permitted have any other seizures in the two hours prior, and seizures that did not meet this condition were excluded from this study. Additionally, if there was a gap in category A data for which no data were recorded, this A data was discarded and the corresponding seizure was not studied.

**Figure 1:**
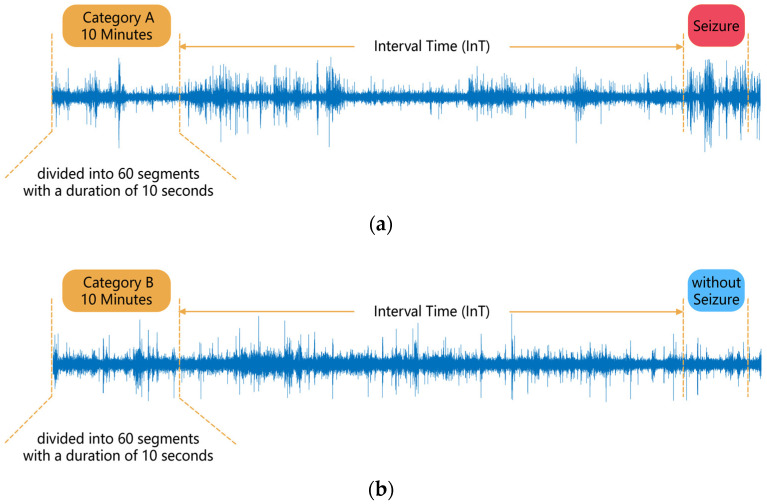
Segmentation of EEG data: (**a**) Category A data, (**b**) Category B data.

Suppose a patient has acquired n segments of category A data as described above, and n segments of category B data were obtained for that patient in order to keep the amount of data consistent between the two categories of EEG. Among all the patient’s EEGs, n sets of 4 consecutive hours without seizures were sought, and 10 min of the first 3 h of each set were randomly selected as a segment of category B data, which ensured that no seizures occurred in each segment of category B data after IT. No gap of no data recording was allowed in each category B data. A 10 min segment of data was then divided into 60 segments of 10 s in length, and the subsequent analysis was carried out in 10 s segments. The data segmentation process is shown in Figure 1.

In some previous studies, researchers have only used the EEG signal from the period before each seizure to analyze and process the signal, for example, defining the first 30 min before a seizure as the pre-ictal period, and the 30–90 min as the interictal period, and identifying the pre-ictal period to predict seizures. In this paper, category A data were obtained in the period before each seizure, while category B data were obtained in the period away from the seizures, which has rarely been used in previous studies, making full use of the patient’s EEG in the dataset. In addition, because the duration of the interictal is longer than the that of the pre-ictal, in some previous studies, the amount of data for the interictal period was higher than the pre-ictal period, which led to class imbalance problems. In this paper, the two categories of data, A and B, which need to be classified and identified, had the same amount of data, avoiding the class imbalance problem.

If the prediction model triggers an alarm, patients can intervene (e.g., take medication) for the upcoming seizure within the InT. Too short an InT may leave patients ill-prepared, while too long may cause stress as patients wait for the upcoming seizure. Therefore, the InT can be set for 30 or 40 min, which gives patients enough time to be well protected and at the same time reduces their anxiety and stress.

After screening, EEG data from 13 patients in the dataset were selected for the experiment. The selected EEG data of all patients contained 23 channels. A total of 39 seizures were selected for the experiment with an InT of 30 min, while 42 seizures were selected with an InT of 40 min. When different values of InT were taken, the amount of category A data for the same patient meeting the above criteria might be different, so that the number of selected seizures appears to be different. Details of the selected patients are shown in Table 1. In the case of patient chb01, with an InT of 30 min, four seizures were selected in the manner described above, so four segments of category A, as well as B EEG data, were divided, each with a recording time of 10 min.

### 2.3. Pre-Processing

EEG signals contains artifacts such as EOG and EMG signals, reducing the signal-to-noise ratio of EEG and, thus, adversely affecting the classification of the two categories of signals [8]. Therefore, a pre-processing method based on CEEMD and wavelet denoising was used for artefact removal.

CEEMD is an adaptive time-frequency analysis method for nonlinear and non-stationary signals that decomposes EEG signals into a number of intrinsic mode functions (IMFs) and a residual signal. The method adds a pair of white noises of opposite amplitude to the source signal as auxiliary noise when decomposing, in order to eliminate the excess auxiliary white noise remaining in the reconstructed signal and to reduce the number of iterations required for the decomposition. The wavelet transform of the EEG signal can be used to obtain a set of wavelet coefficients, where the larger coefficients correspond to the EEG signal and the smaller coefficients correspond to the noise.

**Figure 2:**
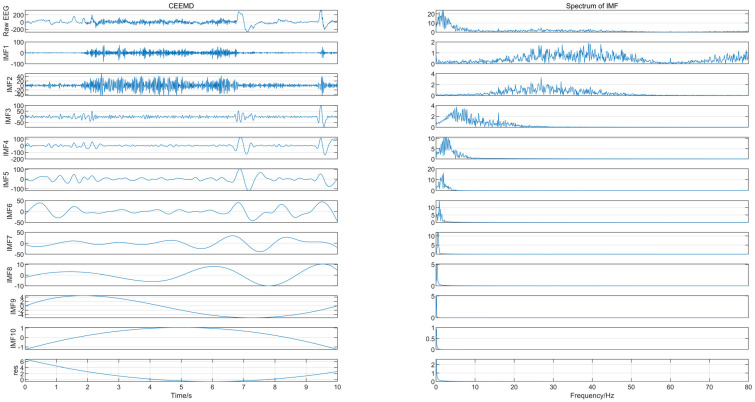
The IMFs and their corresponding spectrum.

**Figure 3:**
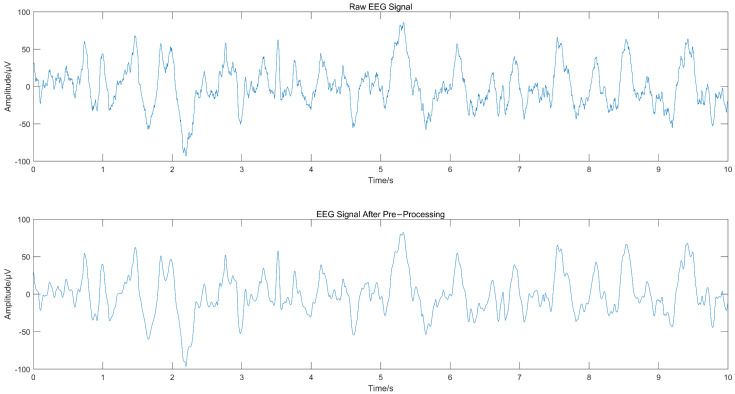
Comparison of EEG signal before and after pre-processing.

The EEG signal was decomposed using CEEMD to obtain several IMFs, and the spectrum corresponding to each IMF was calculated, as shown in Figure 2, from which we can see that IMF1 and IMF2 contained frequency components greater than 30 Hz. Since the frequency of EEG usually lies in the range of 0.5–30 Hz, IMF1 and IMF2 were considered to contain noise. The “db4” wavelet and 4-layers decomposition were used for IMF1 and IMF2 for wavelet denoise with “sqtwolog” threshold method and soft threshold function. IMF1 and IMF2 after denoising were reconstructed with the remaining IMFs (IMF3-IMF10) to obtain the denoised EEG signals. The IMFs containing noise were not discarded directly but were used again for signal reconstruction after wavelet denoising, which avoided excessive loss of useful signal while removing noise. Figure 3 shows the EEG signal before and after pre-processing, with a signal duration of 10 s. The noise of high frequency in the EEG was suppressed without excessive loss of information.

### 2.4. Feature Extraction

The term “entropy” was originally derived from thermodynamics and was first proposed by Clausius in 1865. To date, entropy theory has been developed for more than 100 years, and several entropy features have become common in the field of EEG signal analysis. Researchers have applied various entropy parameters to the prediction of epilepsy seizures and achieved some results [9,10,11].

#### 2.4.1. Entropy Based on Time Domain: Approximate Entropy, Sample Entropy, Permutation Entropy

Approximate entropy (ApEn) is used to describe the unpredictability or randomness of a finite-length signal and is an important nonlinear signal feature. The computation involves embedding a signal into phase space, and estimates the rate of increment in the number of phase space patterns within a predefined value, r, when the embedding dimension of phase space increases from m to m+1 [12]. For a time series x(i) (1 ≤ i ≤ N) of finite length, N, to reconstitute the N − m+1 vectors $X_{m}\left(i\right)$, follow the form:

(1) $$X_{m}\left(i\right)=\left\{x\left(i\right),\;x\left(i+1\right),\;\ldots \;x\left(i+m\;-\;1\right)\right\},\;i=1,2,\;\ldots \;N\;-m+1$$

where m is the embedding dimension. Let $C_{i}^{m}\left(r\right)$ be the probability that any vector $X_{m}\left(j\right)$ is within r of $X_{m}\left(i\right)$. It can be defined as:

(2) $$C_{i}^{m}\left(r\right)=\frac{1}{N\;-m+1}\sum_{j=0}^{N\;-m+1}\Theta \left(d_{\mathrm{ij}}^{m}-r\right),\;i,\;j=1,2,\ldots \;N\;-m+1$$

where d is the distance between vector $X_{m}(j)\;$ and $X_{m}\left(i\right)$, defined as:

(3) $$d_{\mathrm{ij}}^{m}=d\left[X_{i}^{m}{,X}_{j}^{m}\right]=\max\left(\left|x\left(i+k\right)-x\left(j+k\right)\right|\right),\;k=0,\;1,\;\ldots ,\;m\;$$

where Θ is the Heaviside function. Define parameter $\Phi^{m}\left(r\right)$ as:

(4) $$\Phi^{m}(r)=(N\;-{m+1)}^{-1}\sum_{i=1}^{N\;-m+1}{\mathrm{lnC}}_{i}^{m}\left(r\right)$$

Change the embedding dimension to m+1, repeat the above steps to calculate$\;\Phi^{m+1}\left(r\right)$:

(5) $$\Phi^{m+1}(r)=(N\;-{m)}^{-1}\sum_{i=1}^{N\;-m}{\mathrm{lnC}}_{i}^{m+1}(r)\;$$

Then the approximate entropy of the signal is defined by:

(6) $${\mathrm{ApEn}(m,\;r,\;N)=\Phi }^{m}(r)\;-\Phi^{m+1}\left(r\right)$$

Approximate entropy is susceptible to data length (N), similar tolerance (r) and the embedding dimension (m). In this paper, we chose m as 2, and r as 0.2 times the standard deviation of the signal.

Sample entropy (SampEn) has a similar meaning to approximate entropy in that it measures the complexity of a time series by measuring the probability of generating a new pattern in the signal. Similar to approximate entropy, the computation of sample entropy first requires calculating$\;C_{i}^{m}\left(r\right)$. When the embedding dimension is m, define a parameter $B^{m}\left(r\right)$ as:

(7) $$B^{m}(r)=(N\;-{m)}^{-1}\sum_{i=1}^{N\;-m}C_{i}^{m}(r)\;$$

Increase the embedding dimension to m+1, define $A^{m}\left(r\right)$ as:

(8) $$A^{m}(r)=(N\;-{m)}^{-1}\sum_{i=1}^{N\;-m}C_{i}^{m}(r)\;$$

Finally, the sample entropy can be estimated by:

(9) $$\mathrm{SampEn}(r,\;m,\;N)=-\ln\frac{A^{m}\left(r\right)}{B^{m}\left(r\right)}\;$$

Permutation entropy (PEn) is an entropy parameter that measures the complexity of a time series and is characterized by simplicity of calculation and resistance to noise interference. Given a time series x(i) (1 ≤ i ≤ N). Firstly, the reconstruction time series:

(10) $$X_{i}=\left\{x\left(i\right),x\left(i+\tau \right),\ldots ,x\left(i+\left(m\;-1\right)\tau \right)\right\},\;i=1,\;2,\;\ldots \;N\;-(m-1)\tau \;$$

where τ is time delay, and m is the embedding dimension.

The elements in each of the reconstructed components $X_{i}$ are rearranged in ascending order:

(11) $$x\left(i+\left(j_{1}-1\right)\tau \right)\leq x\left(i+\left(j_{2}-1\right)\tau \right)\leq \ldots \leq x\left(i+\left(j_{m}-1\right)\tau \right)\;$$

where $j_{1},\;j_{2},\ldots \;j_{m}$ denotes the index of the column in which each element of the reconstructed component is located. As a result, each reconstructed component of a time series yields a sequence of symbols $j_{1},\;j_{2},\ldots \;j_{m}$. There are m! different sequences in the m-dimensional phase space; let the number of occurrences of the jth sequence be $n_{j}$, then its probability of occurrence $p_{j}$ can be calculated by:

(12) $$p_{j}=\frac{n_{j}}{\sum_{j=1}^{m!}n_{j}}$$

According to the formula of Shannon entropy, there are:

(13) $$H_{x}(m)=-\sum_{j=1}^{m!}p_{j}{\mathrm{lnp}}_{j}\;$$

Finally, the permutation entropy can be estimated by:

(14) $$\mathrm{PEn}=\frac{H_{x}\left(m\right)}{\ln(m!)}$$

#### 2.4.2. Entropy Based on Frequency Domain: Spectral Entropy

Spectral entropy (SpEn) is the Shannon entropy of the power spectral density of the signal [13]. It is calculated as follows:

(15) $$\mathrm{SpEn}=-\sum_{j=1}p_{f}{\mathrm{logp}}_{f}\;$$

where $p_{f}$ is the relative power of the component with frequency f.

#### 2.4.3. Entropy Based on Time-Frequency Domain: Wavelet Entropy

Wavelet decomposition is performed on a segment of the signal with a scale of j and a window length of$\;N_{j}$, then the wavelet energy $E_{j}^{\left(N_{j}\right)}$ at each scale j is defined as:

(16) $$E_{j}^{\left(N_{j}\right)}=\sum_{k}d_{j}{\left(k\right)}^{2}\;$$

where $d_{j}\left(k\right)$ are the wavelet coefficients. The total energy of all scales is:

(17) $$E_{\mathrm{tot}}=\sum_{j}E_{j}^{\left(N_{j}\right)}\;$$

The percentage of $E_{j}^{\left(N_{j}\right)}$ in $E_{\mathrm{tot}}$ is:

(18) $$p_{j}^{\left(N_{j}\right)}=\frac{E_{j}^{\left(N_{j}\right)}}{E_{\mathrm{tot}}}\;$$

Finally, the wavelet entropy of the signal is calculated as follows:

(19) $$\mathrm{WEn}=-\sum_{j}p_{j}^{\left(N_{j}\right)}{\mathrm{logp}}_{j}^{\left(N_{j}\right)}\;$$

The five entropy parameters described above were calculated for each of the 23 channels for each 10 s segment of data for all patients. Assuming a patient had a total of n selected seizures, then they had 2n segments of category A and B data. The feature extraction process generated a feature matrix of 2n × 60 rows and 23 × (5+1) columns, where the last column was labeled as the target value of 1 for category A data, and 0 for category B. For example, the feature extraction for patient chb01 resulted in a feature matrix of size 480 × 116.

### 2.5. Classification

Ensemble learning is the process of constructing and combining multiple learners to accomplish specific learning tasks. The general structure is to generate a set of “base learners”, which are then combined with a certain strategy to produce the final result. Based on the way base learners are generated, there are two broad categories of ensemble learning methods: bagging, in which the base learners are built in parallel and do not affect each other, and boosting, in which the base learners are built one by one and the previous base learner has an impact on the creation of the next. Ensemble learning methods based on boosting (e.g., AdaBoost, GBDT, XGBoost, etc.) have been widely used in many fields.

**Figure 4:**
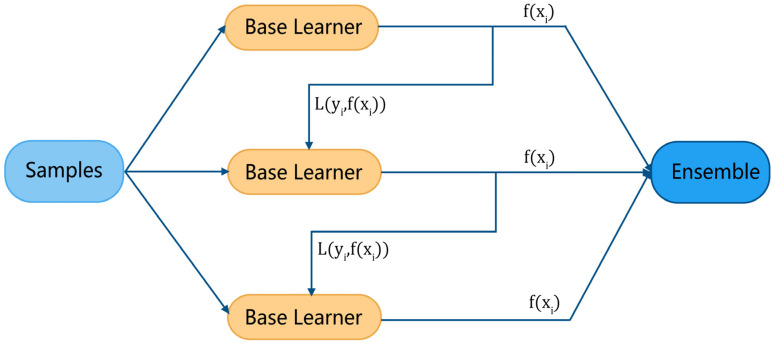
Boosting algorithm modeling process.

Figure 4 illustrates the general modeling process of boosting. For each sample in the training set$\;{(x}_{i}{,y}_{i})$ (where $x_{i}$ is the feature vector and$\;y_{i}$ is the target), the base learner gives its evaluation result $f\left(x_{i}\right)$ and calculates a loss function ${L(y}_{i},f\left(x_{i}\right))$, then, adaptively influences the construction of the next base learner by a certain rule. Finally, the evaluation results of the ensemble learner are derived from all base learners.

Assuming that the evaluation result of sample $x_{i}$ under the tth base learner is $f_{t}\left(x_{i}\right)$ and a total of T base learners are constructed, the output result $H\left(x_{i}\right)$ of the boosting ensemble learner is the weighted sum of all base learners, which can be expressed as:

(20) $$H\left(x_{i}\right)=\sum_{t=1}^{T}\phi_{t}f_{t}\left(x_{i}\right)$$

where $\phi_{t}$ denotes the weight of the tth base learner. It is worth noting that the final evaluation result is not calculated after all the base learners are constructed, but is computed over iterations as the base learners are constructed. That is, after the first t base learner is constructed, there are:

(21) $$H_{t}\left(x_{i}\right){=H}_{t-1}\left(x_{i}\right)+\phi_{t}f_{t}\left(x_{i}\right)$$

GBDT is one of the most representative algorithms in boosting and one of the most consistently performing machine learning algorithms in practical application scenarios. GBDT uses CART decision trees as base learners and features negative gradients of the loss function of each base learner as residuals, and builds the base learners by continuously fitting the residuals. For the sample ${(x}_{i}{,y}_{i})$, the tth base learner evaluates the result of $f_{t}\left(x_{i}\right)$, the loss function is ${L(y}_{i}{,f}_{t}\left(x_{i}\right))$, then the residual is the negative gradient of the loss function, i.e.,

(22) $$r_{\mathrm{it}}=-{\left[\frac{\partial {L(y}_{i}{,\;f}_{t}{(x}_{i}))}{\partial f_{t}{(x}_{i})}\right]}_{f_{t}{(x}_{i}{)=f}_{t-1}{(x}_{i})}\;$$

Use the residuals to replace the labels of the samples to obtain new samples ${(x}_{i}{,r}_{\mathrm{it}})$, then use the new samples to construct the next base learner. This step is repeated until the number of base learners reaches the preset value.

(23) $${L(y}_{i}{,\;f}_{t}{(x}_{i}))=-{(y}_{i}{\mathrm{logp}(x}_{i})+(1-y_{i})\log(1-{p(x}_{i})))\;$$

where ${p(x}_{i})$ is the result of the evaluation based on the first t evaluation result of the base learner $H_{t}\left(x_{i}\right)$ and the probability value calculated by the Sigmoid function, i.e.,

(24) $$p\left(x_{i}\right){=\mathrm{Sigmoid}(H}_{t}\left(x_{i}\right))\;$$

According to Equation (21), the first base classifier is constructed with:

(25) $$H_{1}\left(x_{i}\right){=H}_{0}\left(x_{i}\right)+\phi_{1}f_{1}\left(x_{i}\right)\;$$

where $H_{0}\left(x_{i}\right)$ is called the “initial result” of GBDT and is often taken as zero. In order to improve the classification effect of GBDT, a random forest (RF) classifier was trained first, and each sample was given a prediction result and its corresponding probability by the RF classifier. This probability was taken as the initial result of GBDT, and then the “RF + GBDT” classification model was constructed.

The feature matrices calculated for all patients in the feature extraction session were fed separately into a GBDT classifier with random forest as the initial result to construct a patient-specific EEG signal classification model. Each 10 s segment of the EEG was a sample, and the output of the classifier resulted in a target value of 0 or 1. Ten-fold cross validation procedures were followed to evaluate the generalization performance of the proposed approach.

### 2.6. Post-Processing

**Figure 5:**
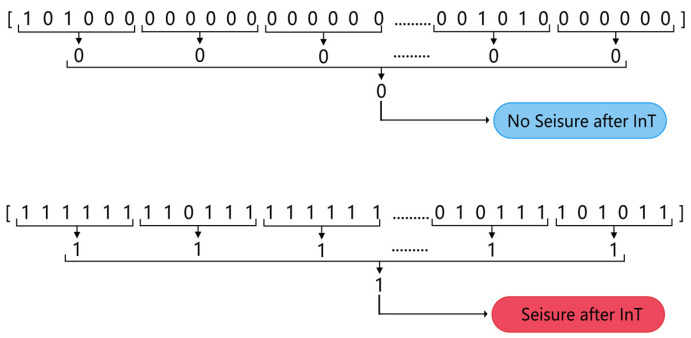
Two-step “k of n” post-processing.

A two-step ‘k of n’ method was used to post-process the classified data to produce the final seizure prediction results while reducing the false alarm rate. For every 10 min segment of EEG data (containing 60 segments of 10 s in length), the classifier output a sequence of 60 numbers with a predictive target of 0 or 1. First, if three or more of the six consecutive numbers in the sequence were 1, then those six numbers were recorded as 1, otherwise they were recorded as 0. Once this step was completed, a sequence of 10 numbers was obtained. Secondly, if 5 or more of the numbers in a sequence of 10 were 1, the 10 numbers was recorded as 1, otherwise 0 was recorded. At this point, a prediction value of 0 or 1 was generated for every 10 min of data. A prediction value of 1 predicted the segment of EEG as category A, where a seizure was considered to occur after a period of time and an alarm was given; conversely, it was predicted as category B, where no seizure was considered to occur after a period of time. Figure 5 illustrates the process of post-processing.

## 3. Results

### 3.1. Evaluation Metrics

Based on the output of the GBDT classifier, the confusion matrix was drawn and used as the basis for calculating accuracy, sensitivity, false positive rate (FPR) and F1-score as the evaluation metrics of the classifier. Equations (26)–(29) are the formulas for the four metrics.

(26) $$\mathrm{Accuracy}=\frac{\mathrm{TP}+\mathrm{TN}}{\mathrm{TP}+\mathrm{TN}+\mathrm{FP}+\mathrm{FN}}$$

(27) $$\mathrm{Sensitivity}=\frac{\mathrm{TP}}{\mathrm{TP}+\mathrm{FN}}$$

(28) $$\mathrm{FPR}=\frac{\mathrm{FP}}{\mathrm{FP}+\mathrm{TN}}$$

(29) $$F1\text{-}\mathrm{score}=\frac{2\mathrm{TP}}{2\mathrm{TP}+\mathrm{FN}+\mathrm{FP}}$$

The post-processing session gave the prediction results for each 10 min data segment. If a segment of category A was correctly predicted, this was considered a successful prediction of a seizure, and conversely, this seizure was considered missed; if a segment of category B was incorrectly predicted as category A, this was considered a false alarm. The number of successful and missed predictions and false alarms were calculated for each patient based on the post-processing results.

### 3.2. Result

#### 3.2.1. Classification Result

The classification evaluation metrics at InT of 30 and 40 min are shown in Table 2 and Table 3, respectively. The average accuracy for both categories of EEG at InT of 30 min was 91.76%, the sensitivity was 91.87%, the FPR was 0.083 and the F1-score was 91.78%. The average accuracy at InT of 40 min was 92.50%, the sensitivity was 91.90%, the FPR was 0.069 and F1-score was 92.37%. Using support vector machines (SVM), random forest (RF) and GBDT with zero initial results as classifier separately, the results (shown in Table 4) indicated that the “RF+GBDT” classification method used in this paper outperformed the above three classifiers.

#### 3.2.2. Prediction Result

The seizure prediction results are shown in Table 5 and Table 6. A total of 39 seizures were selected at an InT of 30 min, and 38 were predicted, with one missed seizure and one false alarm. A total of 42 seizures selected were successfully predicted at an InT of 40 min, with one false alarm. The results show that the proposed method can provide accurate early prediction of seizures with a low number of false alarms.

## 4. Discussion

### 4.1. Comparison with Other Approaches

Table 7 shows the comparison between the proposed seizure prediction method and some of the existing methods, all of which use data from the CHB-MIT Scalp EEG Database. In comparison, the method proposed in this paper outperformed some of the existing methods and was slightly inferior to some methods that use deep learning.

### 4.2. Practical Application

In this paper, an epilepsy prediction method based on the nonlinear features of EEG signal and gradient boosting decision tree was proposed. The experimental results illustrate that this method can effectively classify EEG signals and achieve early prediction of seizures with a low false alarm rate. This method can provide early warning of seizures and reduce the harm suffered by epilepsy patients, which has some practical application prospects.

Patients can have sufficient EEG signals collected in hospitals and other institutions, and their EEG are subjected to a series of analyses and processing by the relevant researchers, including data segmentation, pre-processing, feature extraction, classification and post-processing, to form a patient-specific seizure prediction model. Once an early alarm is issued by the model, patients, their families or healthcare professionals can intervene in a timely manner (with medication or electrical stimulation, etc.) to prevent the seizure or reduce its severity, thus, greatly reducing the physical and psychological harm suffered by the patient.

In order to achieve the practical application of prediction models, the algorithms used in the models must have the ability of strong real-time analysis, which requires the design of algorithms with low complexity, short computing time and high accuracy. Making more accurate predictions with fewer features, speeding up the computing time of classifiers, and promoting the practical application of this approach will be future research directions.

### 4.3. Limitation of the Current Study

In this paper, a series of analyses of EEG signals from epileptic patients in the CHB-MIT Scalp EEG Dataset, including pre-processing, feature extraction and classification, were carried out to predict epilepsy seizures, early. However, there are some limitations to our current study.

First, the seizure prediction approach proposed in this paper relied on the annotation of seizures in the dataset. As described in Section 2.2, the 10 min prior to the InT before a certain seizure, was divided into category A data. In other words, the acquisition of category A data relied on the annotations about seizures, which also makes this approach still somewhat far from practical application. If the annotations of seizures could be ignored, and a more comprehensive analysis of the patient’s EEG could be carried out to identify the pattern of changes in EEG prior to seizures in each patient, it would be possible to predict whether a seizure will occur for any segment of the EEG without annotation. The ultimate goal of this and related studies is to analyze the EEG of diagnosed patients in order to predict seizures early and help them improve their quality of life. To achieve this, it is necessary to address the limitation of relying on annotations.

Second, it is still unknown what impact the choice of InT will have on the performance of the model. In this paper, the InT was only set to 30 and 40 min and no further analysis of the impact of the InT was undertaken. As described in Section 2.2, the choice of InT in this paper was made in order to give patients sufficient time to take the intervention without increasing their anxiety. From a practical application point of view, our current study prefers to improve the model performance as much as possible with a fixed InT. However, it is undeniably meaningful to analyze the impact of InT. For example, will a further increase in InT invalidate the model? Is there a threshold value of InT at which the model performs best? Addressing these issues will further refine the prediction model.

## 5. Conclusions

In this paper, a method based on nonlinear features of EEG signals and GBDT was proposed for early prediction of epilepsy seizures. To avoid the class imbalance problem, the EEG signals were divided into two categories: with and without seizures after a period of time. Based on CEEMD and wavelet denoising, the two categories of EEG signals were pre-processed to remove noise. Then, approximate entropy, sample entropy, permutation entropy, spectral entropy and wavelet entropy features were calculated; and a GBDT classifier with random forest as the initial result was used to classify the two categories of EEG. Finally, a two-step “k of n” method was used to obtain the prediction results. The proposed method was evaluated on the CHB-MIT Scalp EEG Database, and the results showed that it could accurately classify the two categories of EEG signals with a low false alarm rate. In addition, our “RF + GBDT” classification method outperformed other traditional machine learning classifiers. The promising results suggest the effectiveness of the proposed approach for early prediction of seizures, which can help patients to intervene in advance, to reduce the harm they suffer. Meanwhile, this approach has some limitations in that it relies on annotations about seizures and the impact of InT on the model is not yet clear. Further study and optimization is warranted.

## Figures and Tables

**Table 1:** Details of selected patients.

Patient ID	Gender *	Age	Number of Total Seizures	Number of Seizures Selected	
				InT = 30 min	InT = 40 min
chb01	F	11	7	4	4
chb02	M	11	3	2	2
chb04	M	22	4	2	2
chb05	F	7	5	2	4
chb06	F	1.5	10	5	5
chb07	F	14.5	3	3	3
chb09	F	10	4	3	3
chb10	M	3	7	4	5
chb12	F	2	40	3	4
chb14	F	9	8	2	2
chb15	M	16	20	5	4
chb22	F	9	3	2	2
chb23	F	6	7	2	2
Total	--	--	121	39	42

* Gender: F (female), M (male).

**Table 2:** Classification evaluation metrics (InT = 30 min).

Patient ID	Acc *	Sen *	FPR *	F1-Score
chb01	99.37%	99.58%	0.008	99.38%
chb02	97.92%	99.17%	0.033	97.94%
chb04	95.83%	95.00%	0.033	95.80%
chb05	97.08%	97.50%	0.033	97.10%
chb06	81.00%	78.67%	0.163	80.68%
chb07	94.72%	96.11%	0.067	94.79%
chb09	95.56%	95.00%	0.039	95.53%
chb10	92.71%	92.92%	0.075	92.72%
chb12	92.78%	93.89%	0.083	92.86%
chb14	76.67%	79.17%	0.258	77.24%
chb15	81.33%	80.67%	0.180	81.21%
chb22	92.50%	92.50%	0.075	92.50%
chb23	95.42%	94.17%	0.033	95.36%
Average	91.76%	91.87%	0.083	91.78%

* Acc: accuracy; Sen: sensitivity; FPR: false positive rate.

**Table 3:** Classification evaluation metrics (InT = 40 min).

Patient ID	Acc *	Sen *	FPR *	F1-Score
chb01	99.79%	100.00%	0.004	99.79%
chb02	95.83%	96.67%	0.050	95.87%
chb04	97.08%	95.83%	0.017	97.05%
chb05	95.83%	97.08%	0.054	95.88%
chb06	81.33%	76.33%	0.137	80.35%
chb07	94.72%	97.22%	0.078	94.85%
chb09	95.56%	96.67%	0.056	95.60%
chb10	93.00%	90.67%	0.047	92.83%
chb12	82.29%	82.92%	0.183	82.40%
chb14	81.67%	77.50%	0.142	80.87%
chb15	97.08%	97.08%	0.029	97.08%
chb22	92.50%	90.83%	0.058	92.37%
chb23	95.83%	95.83%	0.042	95.83%
Average	92.50%	91.90%	0.069	92.37%

* Acc: accuracy; Sen: sensitivity; FPR: false positive rate.

**Table 4:** Comparison of evaluation metrics of different classifiers.

Classification Method	InT = 30 min			InT = 40 min		
	Acc *	Sen *	FPR *	Acc *	Sen *	FPR *
SVM	89.41%	88.33%	0.095	89.66%	85.62%	0.063
RF	90.55%	90.55%	0.094	91.45%	90.77%	0.079
$\mathrm{GBDT}\;(H_{0}\left(x_{i}\right)=0$)	90.33%	90.18%	0.095	91.15%	90.68%	0.084
RF + GBDT	92.00%	91.87%	0.083	92.50%	91.90%	0.069

* Acc: accuracy; Sen: sensitivity; FPR: false positive rate.

**Table 5:** Prediction results (InT = 30 min).

Patient ID	Number of Seizures	Number of Predictions	Number of Missed Predictions	Number of False Alarms
chb01	4	4	0	0
chb02	2	2	0	0
chb04	2	2	0	0
chb05	2	2	0	0
chb06	5	5	0	0
chb07	3	3	0	0
chb09	3	3	0	0
chb10	4	4	0	0
chb12	3	3	0	0
chb14	2	2	0	0
chb15	5	4	1	1
chb22	2	2	0	0
chb23	2	2	0	0
Total	39	38	1	1

**Table 6:** Prediction results (InT = 40 min).

Patient ID	Number of Seizures	Number of Predictions	Number of Missed Predictions	Number of False Alarms
chb01	4	4	0	0
chb02	2	2	0	0
chb04	2	2	0	0
chb05	4	4	0	0
chb06	5	5	0	0
chb07	3	3	0	0
chb09	3	3	0	0
chb10	5	5	0	0
chb12	4	4	0	1
chb14	2	2	0	0
chb15	4	4	0	0
chb22	2	2	0	0
chb23	2	2	0	0
Total	42	42	0	1

**Table 7:** Comparison of existing methods.

Authors	Year	Classifier	Acc *	Sen *	FPR *
Alotaiby et al. [14]	2017	LDA	-	89%	0.390
Agboola et al. [15]	2019	SVM	-	87.26%	0.080
Zhang et al. [16]	2021	Bi-LSTM	80.09%	-	0.260
Rusnac et al. [17]	2019	MLP	91.14%	91.37%	0.090
Daoud et al. [18]	2019	DCAE + Bi-LSTM + CS	99.66%	99.72%	0.004
Jana et al. [19]	2021	CNN	99.47%	97.83%	0.0764
Yan et al. [20]	2022	Transformer	-	96.01%	0.047
This work (InT = 30 min)	-	RF + GBDT	91.76%	91.87%	0.083
This work (InT = 40 min)	-	RF + GBDT	92.50%	91.90%	0.069

* Acc: accuracy; Sen: sensitivity; FPR: false positive rate.

## Data Availability

The data presented in this study are openly available in CHB-MIT Scalp EEG Database at (https://physionet.org/content/chbmit/1.0.0/, accessed on 10 March 2022), reference number [7].
